# Associations of maternal iron deficiency with malaria infection in a cohort of pregnant Papua New Guinean women

**DOI:** 10.1186/s12936-022-04177-8

**Published:** 2022-05-26

**Authors:** Holger W. Unger, Andie Bleicher, Maria Ome-Kaius, Elizabeth H. Aitken, Stephen J. Rogerson

**Affiliations:** 1 Department of Obstetrics and Gynaecology, Royal Darwin Hospital, Darwin, NT Australia; 2 Menzies School of Health Research, Charles Darwin University, Darwin, NT Australia; 3 Department of Clinical Sciences, Liverpool School of Tropical Medicine, Liverpool, UK; 4 Department of Medicine (RMH), Peter Doherty Institute for Infection and Immunity, The University of Melbourne, Melbourne, VIC Australia; 5 Papua New Guinea Institute of Medical Research, Goroka, Papua New Guinea; 6 Department of Infectious Diseases, Peter Doherty Institute for Infection and Immunity, The University of Melbourne, Melbourne, VIC Australia; 7 Department of Microbiology and Immunology, Peter Doherty Institute for Infection and Immunity, The University of Melbourne, Melbourne, VIC Australia

**Keywords:** Iron deficiency, Iron supplementation, *Plasmodium falciparum*, *Plasmodium vivax*, Risk

## Abstract

**Background:**

Iron deficiency (ID) is common in malaria-endemic settings. Intermittent preventative treatment of malaria in pregnancy (IPTp) and iron supplementation are core components of antenatal care in endemic regions to prevent adverse pregnancy outcomes. ID has been associated with reduced risk of malaria infection, and correspondingly, iron supplementation with increased risk of malaria infection, in some studies.

**Methods:**

A secondary analysis was conducted amongst 1888 pregnant women enrolled in a malaria prevention trial in Papua New Guinea. Maternal ID was defined as inflammation-corrected plasma ferritin levels < 15 μg/L at antenatal enrolment. Malaria burden (*Plasmodium falciparum*, *Plasmodium vivax*) was determined by light microscopy, polymerase chain reaction, and placental histology. Multiple logistic and linear regression analyses explored the relationship of ID or ferritin levels with indicators of malaria infection. Models were fitted with interaction terms to assess for modification of iron-malaria relationships by gravidity or treatment arm.

**Results:**

Two-thirds (n = 1226) and 13.7% (n = 258) of women had ID and peripheral parasitaemia, respectively, at antenatal enrolment (median gestational age: 22 weeks), and 18.7% (120/1,356) had evidence of malaria infection on placental histology. Overall, ID was associated with reduced odds of peripheral parasitaemia at enrolment (adjusted odds ratio [aOR] 0.50; 95% confidence interval [95% CI] 0.38, 0.66, P < 0.001); peripheral parasitaemia at delivery (aOR 0.68, 95% CI 0.46, 1.00; P = 0.050); and past placental infection (aOR 0.35, 95% CI 0.24, 0.50; P < 0.001). Corresponding increases in the odds of infection were observed with two-fold increases in ferritin levels. There was effect modification of iron-malaria relationships by gravidity. At delivery, ID was associated with reduced odds of peripheral parasitaemia amongst primigravid (AOR 0.44, 95% CI 0.25, 0.76; P = 0.003), but not multigravid women (AOR 1.12, 95% CI 0.61, 2.05; P = 0.720). A two-fold increase in ferritin associated with increased odds of placental blood infection (1.44, 95% CI 1.06, 1.96; P = 0.019) and active placental infection on histology amongst primigravid women only (1.24, 95% CI 1.00, 1.54; P = 0.052).

**Conclusions:**

Low maternal ferritin at first antenatal visit was associated with a lower risk of malaria infection during pregnancy, most notably in primigravid women. The mechanisms by which maternal iron stores influence susceptibility to infection with *Plasmodium* species require further investigation.

*Trial registration*

**Supplementary Information:**

The online version contains supplementary material available at 10.1186/s12936-022-04177-8.

## Background

Malaria in pregnancy continues to be a major global health issue. The principal malaria species infecting pregnant women are *Plasmodium falciparum* and *Plasmodium vivax*. In 2018, approximately 11 million women were exposed to *Plasmodium* spp*.* infection (primarily *P. falciparum*) in moderate to high transmission areas in sub-Saharan Africa alone [1]. A much greater number of pregnancies are at risk of infection when areas of lower transmission are considered, including malaria-endemic settings in the Americas and the Asia–Pacific region, where *P. vivax* is often endemic [2]. Malaria is a principal cause of maternal anaemia and maternal death, causes preterm birth and fetal growth restriction, and increases the risk of fetal loss (miscarriage, stillbirth) and neonatal death [3]. In sub-Saharan Africa, at least one in five cases of low birth weight (birthweight < 2,500 g) are attributable to MIP [1], and low birth weight infants may be at risk of long-term health sequelae, based on studies from elsewhere [4].

A hallmark of *P. falciparum* infection in pregnancy is placental sequestration. Infected erythrocytes bind to chondroitin sulphate A present on the surface of the syncytiotrophoblast lining the placental intervillous space, thereby avoiding splenic clearance. This process is mediated by the parasite antigen VAR2CSA [5]. If untreated, placental malaria triggers a cascade of inflammatory processes that leads to impairments in placental development and transplacental nutrient transfer [5]. In areas of moderate to high transmission intensity, women with placental infections are frequently asymptomatic and aparasitaemic, and the risk of placental malaria falls with increasing gravidity [5]. At least half of placental infections are missed by current point-of-care tests [6]. Monthly intermittent preventative treatment in pregnancy (IPTp) with sulfadoxine-pyrimethamine (SP) is currently recommended in malaria-endemic regions of sub-Saharan Africa to treat occult placental infections and provide protection from new infections [7].

Iron deficiency (ID) in pregnancy is common in malaria-endemic settings. ID is independently associated with maternal anaemia, fetal growth restriction and low birth weight [8]. The World Health Organization (WHO) recommends routine iron supplementation during pregnancy in populations with a prevalence of anaemia ≥ 40%, on the proviso that iron supplementation is combined with adequate malaria prevention measures in endemic areas [9]. This advice is based on concerns over reports of increased risk of malaria infection in supplemented women and children [10–14]. Correspondingly, ID has been associated with reduced risk of malaria infection in children [10] and pregnant women [13, 15–18]. Significant heterogeneity between studies exists, with a number of studies reporting no association of estimates of maternal iron stores or iron supplementation with malaria in pregnancy [13, 19, 20]. Iron supplementation in pregnancy was not associated with increased risk of malaria infection, including placental infection, in two randomized trials in Kenya and Tanzania conducted amongst women concurrently using bed nets and SP-IPTp [21, 22].

In Papua New Guinea (PNG), ID in pregnancy is common in malaria-endemic areas [23, 24]: nearly two-thirds of women are iron deficient. Daily iron supplementation in combination with monthly IPTp with SP during pregnancy is national policy. Iron supplementation studies conducted in PNG in the 1980s first highlighted the potential existence of iron-malaria interactions [10, 11].

The aim of the present analysis was to determine the relationship of maternal iron deficiency at first antenatal visit with malaria infection (*P. falciparum* and/or *P. vivax*) during pregnancy, and to assess whether these associations are modified by gravidity and malaria prevention strategy, in a large cohort of pregnant Papua New Guinean women.

## Methods

A total of 2793 women were randomized to monthly IPTp with SP plus azithromycin (AZ) or a single course of SP plus chloroquine (CQ) at antenatal enrolment in the PNG-IPTp trial (NCT01136850). The details of the trial are described in detail elsewhere [25]. Drawing on routinely collected samples and data from the PNG-IPTp trial a secondary analysis to assess relationships between ID and ferritin levels at antenatal enrolment with malaria infection in pregnancy was conducted. The analysis considered women who had a singleton live birth (no detectable congenital anomaly), and for whom data on ferritin levels and peripheral malaria infection at enrolment, and malaria infection at delivery by light microscopy (LM) and quantitative real-time polymerase chain reaction (qPCR) was complete.

### Study area and population

Women in the PNG-IPTp trial were recruited at clinics, health centres, and hospitals located in Madang and Sumkar districts of Madang Province on the North Coast of PNG from 2009–2013, as previously described [25]. The study area is characterized by perennial transmission of moderate intensity of both *P. falciparum* and *P. vivax* [26]. Maternal ID and anaemia are common [24, 27]. *P. falciparum* was the predominant malaria species detected in pregnant women during the study period [28]. Women were screened when attending for their first antenatal visit and enrolled in the parent trial when all inclusion criteria were met and written informed consent was provided. Key inclusion criteria included an estimated gestational age < 27 weeks at first antenatal visit; singleton pregnancy; no prior administration of IPTp; and absence of chronic medical conditions in the mother [25]. The clinical trial and secondary analyses relating to maternal malaria infection and anaemia, including the present study, were approved by the PNG Institute of Medical Research (PNGIMR) Institutional Review Board (0815), the PNG Medical Research Advisory Council (08.01), and the Melbourne Health Human Research Ethics Committee (2008.162).

### Clinical assessments and sample collection

Key demographic and clinical characteristics were determined at enrolment and collected alongside venous blood samples for malariometrics, haemoglobin (Hb) measurements (HemoCue Ltd, Angelholm, Sweden, accuracy 1 g/L), and measurement of ferritin and inflammatory markers [24]. Women were provided with insecticide-treated bed nets and randomized 1:1 to monthly IPTp with SP (1500/75 mg) plus AZ (1 g twice daily for 2 days) or a single course of SP plus chloroquine (450 to 600 mg, daily for 3 days) at their first antenatal visit. Iron-folate supplementation (one tablet of ferrous sulphate 270 mg [87.4 mg elemental iron] plus 400 μg folic acid) was offered routinely and women were advised to take one tablet daily. Women who underwent Hb testing that identified a Hb ≤ 90 g/L were recommended to take two iron-folate tablets and in addition were provided with albendazole, and treatment response was assessed a month later. Iron-folate supplementation during pregnancy was not monitored. Women diagnosed with anaemia after enrolment were retained in the parent trial, and there was no difference in the prevalence of anaemia at delivery between trial arms [25]. Clinical data and blood samples were also collected when practical at subsequent scheduled and unscheduled antenatal visits. At delivery, when women were able to deliver at a participating health centre or hospital, peripheral and placental blood samples and placental biopsies were taken and Hb was measured again [29]. Ferritin was measured at enrolment only. Placental blood was collected by incision in the maternal surface of the placenta and aspiration of blood welling from the incision.

### Laboratory analyses

Peripheral and placental blood samples were screened for *P. falciparum* and *P. vivax* infection by light microscopy and polymerase chain reaction (using a duplex qPCR that identified each species) [28]. Histological analysis of placental biopsies was performed in accordance with established methodology [30, 31] and reported as active infection (presence of parasites [acute infection] ± pigment in monocytes and/or fibrin [chronic infection]), past infection (pigment only), and no infection.

Maternal iron stores were estimated using ferritin concentrations measured in enrolment plasma by an enzyme-linked immunosorbent assay (ELISA) developed by our group [32]. Ferritin is an iron storage protein which is expressed in response to intracellular iron concentrations [33]. Plasma ferritin reflects overall storage iron, can be easily measured, and correlates with reference methods for the diagnosis of ID such as bone marrow aspiration. However, ferritin is an acute phase reactant and plasma levels increase during systemic inflammation. To facilitate adjustments of ferritin for concurrent inflammation, as recommended by the WHO [34], concentrations of C-reactive protein (CRP) and α-1-acid glycoprotein (AGP) were measured by ELISA using Human Quantikine ELISA kits (R&D Systems, Minneapolis, MN, USA).

### Definitions

ID was defined as a plasma ferritin level < 15 μg/L at antenatal enrolment [35]. ID and ferritin (transformed to log base 2 [i.e. log_2_] ferritin for ease of interpretation) were used as exposure measures. Ferritin levels were corrected for inflammation using the ‘internal regression correction’ method devised by Namaste et al. [36], and adjusted for CRP, AGP, and concurrent malaria parasitaemia [21]. Additional correction for malaria parasitaemia (infection present or absent according to LM and/or qPCR at the time of ferritin measurement) was considered necessary as, contrary to findings by Namaste et al., foregoing this would have led to substantial misclassification of iron deficient women as iron replete (i.e. ≥ 15 μg/L) in our cohort: 48.1% of women were classified as ID using the standard correction (CRP and AGP), compared to 64.9% when correcting for CRP, AGP and parasitaemia). Internal reference values, i.e. threshold values above which ferritin adjustments are assumed to be required, were 0.2 mg/L and 87 mg/L for CRP and AGP, respectively. Regression coefficients are reported in Additional file 1.

Outcome measures included peripheral malaria infection (*P. falciparum* or *P. vivax*) by light microscopy and/or qPCR at enrolment or delivery; placental blood infection by light microscopy and/or qPCR; and active or past infection on placental histology [29].

## Statistical analyses

Associations of ID or ferritin (log_2_) at enrolment with indicators of malaria infection at enrolment and delivery were assessed using multiple logistic or linear regression, as appropriate.

Multiple regression adjusted a priori for previously identified predictors of malaria infection in the cohort, including primigravidity, rural residence, maternal age, and trial treatment arm [24, 25, 29]. Models also included gestational age at ferritin measurement as a covariate, to account for physiological depletion of iron stores with advancing gestation. Presence of modification of the association between ID and malaria infection by gravidity (primigravid versus multigravid) was assessed by fitting models with interaction terms between ID and gravidity, or between ID and treatment arm. Presence of modification of the association between ID and malaria infection by enrolment Hb ≤ 90 g/L, a trigger for enhanced iron-folate supplementation, was also assessed for women with complete data. P-values for effect modification were derived from likelihood ratio tests comparing models with and without the interaction terms. A P-value < 0.05 was considered statistically significant. Statistical analyses were performed using Stata version 16.0 (StataCorp, College Station, TX, USA).

## Results

**Fig. 1:**
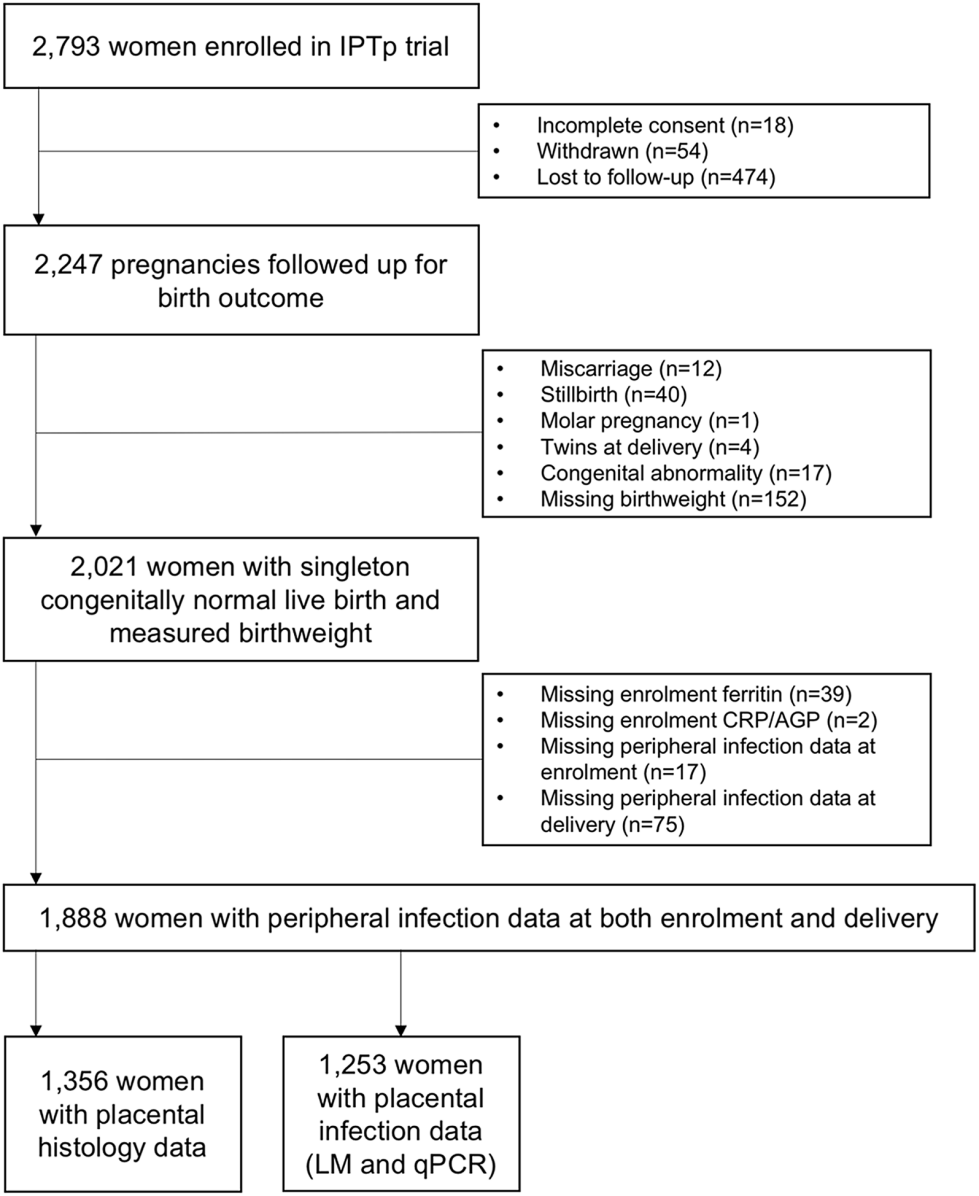
Participant flow chart. CRP, C-reactive protein; AGP, α-1-acid glycoprotein, LM, light microscopy, qPCR, polymerase chain reaction

The analysis included a total of 1,888 women (Fig. 1). The median gestational age at enrolment was 22 gestational weeks (interquartile range [IQR] 19, 24 gestational weeks). Nearly half of the women (n = 931) were primigravid, and the median maternal age was 24 years (IQR 20–28 years) (Table 1). Approximately two-thirds of women resided in rural areas (n = 1239), and 77% of women reported using a bed net at enrolment. A total of 945 women (50.1%) were randomized to SPAZ, and 943 (49.9%) women were randomized to SPCQ. The mean haemoglobin concentration at enrolment was 97 g/L (standard deviation [SD] 15 g/L), and 527 of 1,812 women (29.1%) had an enrolment Hb ≤ 90 g/L. A total of 1,226 women (64.9%) were classified as ID at antenatal enrolment. The median (IQR) ferritin concentration at first antenatal visit was 10.2 μg/L (IQR 5.7–20.4 μg/L).

**Table 1:** Association of malaria infection at first antenatal visit with maternal socio-demographic characteristics, Madang, Papua New Guinea, 2009–2013

Characteristic	All women (n = 1888)	Malaria infection (n = 258)	No malaria infection (n = 1630)
Gestational age (weeks)	22 (19, 24)	21 (18, 24)	22 (20, 24)
Maternal age (years)	24 (20, 28)	23 (20, 27)	24 (20, 28)
Residence			
Rural	1239 (65.6)	204 (79.1)	1035 (63.5)
Urban/peri-urban	649 (34.4)	54 (20.9)	595 (36.5)
Gravidity			
Primigravid	931 (49.3)	139 (53.9)	792 (48.6)
Multigravid	957 (50.7)	119 (46.1)	838 (51.4)
Bed net use			
Yes	1453 (77.0)	199 (77.1)	1254 (76.9)
No	435 (23.0)	59 (22.9)	376 (23.1)
Haemoglobin (g/L)^a^	97 (15)	92 (16)	98 (14)

Mean [standard deviation]; or median (interquartile range); or N (%). Malaria infection at first antenatal visit was defined as presence of *Plasmodium* spp. detected by light microscopy and/or qPCR of peripheral blood

^a^ Data available for 1812 women (245 women with malaria infection)

### Malaria burden

A total of 258 women (13.7%) were parasitaemic at antenatal enrolment. Amongst participants with parasitaemia, 73.6% (n = 190) had *P. falciparum* mono-infections, 4.7% (n = 12) had mixed *P. falciparum*/*P. vivax* infections, 20.2% (n = 52) had *P. vivax* infections, with the remainder having *Plasmodium ovale* or *Plasmodium malariae* infections (n = 4). Half of these infections were submicroscopic (n = 134) (Table 2).

**Table 2:** Malaria infection at first antenatal visit and at birth, by presence or absence of iron deficiency (plasma ferritin), Madang, Papua New Guinea, 2009–2013

	All women	Iron-deficient (ferritin < 15 μg/L)	Iron-replete (ferritin ≥ 15 μg/L)	aOR (95% CI)	P value
First antenatal visit					
Parasite detection	n = 1888	n = 1226	n = 662		
Microscopic	124 (6.6)	47 (3.8)	77 (11.6)	0.30 (0.21, 0.46)	< 0.001
Submicroscopic	134 (7.1)	76 (6.2)	58 (8.8)	0.74 (0.51, 1.07)	0.11
No infection	1630 (86.3)	1103 (90.0)	527 (79.6)	Reference	
Plasmodium species					
* P. falciparum*	190 (10.1)	76 (6.2)	114 (17.2)	0.35 (0.26, 0.49)	< 0.001
* P. falciparum*/*P. vivax*^*a*^	12 (0.6)	6 (0.5)	6 (0.9)		
* P. vivax*	52 (2.8)	39 (3.2)	13 (2.0)	1.76 (0.91, 3.41)	0.093
*P. malariae/ovale*	4 (0.2)	2 (0.2)	2 (0.3)	–	
No infection	1630 (86.3)	1103 (90.0)	527 (79.6)	Reference	
Delivery					
*Peripheral infection*	n = 1888	n = 1226	n = 662		
Parasite detection					
Microscopic	59 (3.1)	36 (2.9)	23 (3.5)	0.97 (0.55, 1.70)	0.91
Submicroscopic	61 (3.2)	30 (2.5)	31 (4.7)	0.48 (0.28, 0.82)	0.008
No infection	1768 (93.6)	1160 (94.6)	608 (91.8)	Reference	
Plasmodium species					
* P. falciparum*	71 (3.8)	40 (3.3)	31 (4.7)	0.77 (0.47, 1.27)	0.31
* P. falciparum*/*P. vivax*^*a*^	3 (0.2)	3 (0.2)	0 (0.0)	–	
* P. vivax*	46 (2.4)	23 (1.9)	23 (3.5)	0.55 (0.30, 1.02)	0.058
No infection	1768 (93.6)	1160 (94.6)	608 (91.8)	Reference	
* Placental infection (blood)*	n = 1253	n = 817	n = 436		
Parasite detection^b^					
Microscopic	40 (3.2)	24 (2.9)	16 (3.7)	1.00 (0.51, 1.99)	0.99
Submicroscopic	23 (1.8)	8 (1.0)	15 (3.4)	0.33 (0.13, 0.83)	0.018
No infection	1190 (95.0)	785 (96.1)	405 (92.9)	Reference	
* Placental infection (histology)*	n = 1356	n = 893	n = 463		
Acute	53 (3.9)	40 (4.5)	13 (2.8)	1.62 (0.83, 3.16)	0.16
Chronic	50 (3.7)	26 (2.9)	24 (5.2)	0.54 (0.30, 0.99)	0.047
Past	151 (11.1)	72 (8.1)	79 (17.1)	0.35 (0.24, 0.51)	< 0.001
No infection	1102 (81.3)	755 (84.6)	347 (75.0)	Reference	

N (%). Analyses adjusted for gravidity, maternal age, malaria chemoprevention regimen, rural location, and gestational age at ferritin measurement. Ferritin levels were adjusted for concurrent inflammation and peripheral *Plasmodium* parasitaemia using the BRINDA (Biomarkers Reflecting Inflammation and Nutritional Determinants of Anemia) approach [36]

aOR: adjusted odds ratio; CI: confidence interval; *P:* *Plasmodium*

^*a*^Mixed *P. falciparum*/*P. vivax* infections were included in regressions models as *P. falciparum* infections

^*b*^Includes 55 *P. falciparum* infections, 1 mixed *P. falciparum*/*P. vivax* infection, and 7 *P. vivax* infections

At delivery, 6.4% of women (n = 120) had malaria parasites detected in peripheral blood: amongst them 59.2% (n = 71) had *P. falciparum* mono-infections, 2.5% (n = 3) had mixed *P. falciparum*/*P. vivax* infections, and 38.3% (n = 46) had *P. vivax* mono-infections. About half of these infections (n = 61) were submicroscopic. Placental blood parasitaemia was detected in 5.0% (n = 63) of women with complete data (n = 1253) for these analyses: 87.3% (55/63) of infections were caused by *P. falciparum* (Table 2). Placental histology data was available for 1,356 study participants. Evidence of infection was detected in 254 (18.7%) of women: amongst them, 40.6% (103/254) had active placental malaria, and 59.4% (151/254) had evidence of past infection (Table 2). One fifth of women (21.4%, 369/1723) had a Hb ≤ 90 g/L at delivery.

### Associations of maternal iron stores and malaria infection during pregnancy

Associations between malaria infection during pregnancy and maternal ID at first antenatal visit are presented in Table 3. ID at antenatal enrolment was associated with reduced odds of concurrent peripheral malaria parasitaemia (adjusted odds ratio [aOR] 0.50; 95% confidence interval [95% CI] 0.38, 0.66, P < 0.001) (Table 3). Specifically, women with ID had reduced odds of *P. falciparum* infection (aOR 0.35, 95% CI 0.26, 0.49; P < 0.001) but not *P. vivax* infection at enrolment (aOR 1.76, 95% CI 0.91, 3.41; P = 0.093) (Table 2). Women with ID had more markedly reduced odds of microscopic (aOR 0.30, 95% CI 0.21, 0.46; P < 0.001) than submicroscopic infection (aOR 0.74, 95% CI 0.51, 1.07; P = 0.11) (Table 2). For a two-fold increase in ferritin, there was a 37% increase in the odds of peripheral parasitaemia at first antenatal visit (aOR 1.37; 95% CI 1.23, 1.53, P < 0.001) (Table 3).

**Table 3:** Association of maternal iron status (ferritin) with malaria infection at antenatal enrolment and at delivery, Madang, Papua New Guinea, 2009–2013

Factor	aOR (95% CI)	P value
Enrolment		
* Peripheral infection*^*a*^		
Iron deficiency		
Ferritin < 15 μg/L	0.50 (0.38, 0.66)	< 0.001
Ferritin ≥ 15 μg/L	Reference	
Log_2_(ferritin)	1.37 (1.23, 1.53)	< 0.001
Delivery		
* Peripheral infection*^*a*^		
Iron deficiency		
Ferritin < 15 μg/L	0.68 (0.46, 1.00)	0.050
Ferritin ≥ 15 μg/L	Reference	
Log_2_(ferritin)	1.27 (1.10, 1.48)	0.001
Placental infection (blood)^a^		
Iron deficiency		
Ferritin < 15 μg/L	0.66 (0.39, 1.14)	0.14
Ferritin ≥ 15 μg/L	Reference	
Log_2_(ferritin)	1.22 (1.00, 1.49)	0.054
Placental infection (histology)^a^		
Iron deficiency		
Active infection	0.91 (0.58, 1.43)	0.68
Past infection	0.35 (0.24, 0.50)	< 0.001
No infection	Reference	
Log_2_(ferritin)		
Active infection	1.04 (0.88, 1.22)	0.64
Past infection	1.65 (1.42, 1.91)	< 0.001
No infection	Reference	

Analyses adjusted for gravidity, maternal age, malaria chemoprevention regimen, rural location, and gestational age at ferritin measurement. Ferritin levels were adjusted for concurrent inflammation and peripheral malaria parasitaemia using the BRINDA (Biomarkers Reflecting Inflammation and Nutritional Determinants of Anemia) approach [36]

aOR: adjusted odds ratio; CI: confidence interval

^a^Include 1,886, 1,886, 1,253 and 1,356 women with malaria infection data (*Plasmodium falciparum*, *P. vivax*) from peripheral blood at enrolment, peripheral blood at delivery, placental blood, and placental histology collected at delivery, respectively

At delivery, when peripheral parasitaemia was less common and proportionally included more *P. vivax* infections, iron-deficient women had reduced odds of peripheral blood infection (aOR 0.68, 95% CI 0.46, 1.00; P = 0.050). Women with ID had reduced odds of peripheral *P. falciparum* infection (aOR 0.77, 95% CI 0.47, 1.27; P = 0.31) and peripheral *P. vivax* infection (aOR 0.55, 95% CI 0.30, 1.02, P = 0.058) (Table 2). Women with ID had reduced odds of peripheral submicroscopic (aOR 0.48, 95% CI 0.28, 0.82; P = 0.008) but not microscopic infection (aOR 0.97, 95% CI 0.55, 1.70; P = 0.91) (Table 2). A two-fold increase in ferritin associated with increased odds of peripheral parasitaemia at delivery (aOR 1.22, 95% CI 1.10, 1.49, P = 0.001; Table 3).

The majority of placental blood infections were caused by *P. falciparum* (Table 2). Women with ID had reduced odds of placental blood infections (aOR 0.66, 95% 0.39, 1.44; P = 0.14); and a corresponding increase in odds of placental infection associated with a two-fold increase in ferritin (aOR 1.22, 95% CI 1.00, 1.49; P = 0.054) (Table 3). Amongst women with histology data, ID at first antenatal visit was associated with reduced odds of past placental infection at delivery (aOR 0.35, 95% CI 0.24, 0.50; P < 0.001) but not with active placental infection (aOR 0.91; 95% CI 0.58, 1.43; P = 0.68) (Table 3). These findings were reciprocated in analyses that assessed the relationship between ferritin levels and malaria infection at delivery, with two-fold increases in ferritin levels associating with increased odds of past infection detected at delivery but not active infection on placental histology (Table 3). However, analyses of associations between ID and active placental infections that assessed subsets of active infection, e.g. acute infection (parasites, no pigment in monocytes or fibrin) or chronic infection (parasites, pigment in monocytes and/or fibrin), indicated reduced odds of chronic placental infection amongst iron-deficient women (Table 2).

Sensitivity analyses were conducted to assess associations between ID and malaria after (a) adjusting ferritin levels for CRP and AGP, but not concurrent malaria infection; and (b) excluding women with malaria parasitaemia at enrolment. The findings were very similar to those presented above (Additional file 1: Tables S1, 2).

### Impact of gravidity or treatment arm on the relationship between antenatal ID and malaria infection at birth

Associations of ID or ferritin levels with malaria infection at delivery were modified by gravidity (Additional file 1: Tables S3). ID was associated with reduced odds of peripheral parasitaemia at birth amongst primigravid (peripheral parasitaemia: AOR 0.44, 95% CI 0.25, 0.76; P = 0.003) but not multigravid women (peripheral parasitaemia: AOR 1.12, 95% CI 0.61, 2.05; P = 0.72) (Additional file 1: Tables S3). This finding was corroborated by a reciprocal increase in the odds of peripheral infection associated with a two-fold increase in ferritin amongst primigravid (aOR 1.51, 95% CI 1.23, 1.84; P < 0.001), but not amongst multigravid women (aOR 1.04, 95% CI 0.83, 1.29); P = 0.76). Likewise, associations of ferritin levels with placental blood infection and acute infection on histology differed by gravidity status. A two-fold increase in ferritin associated with increased odds of placental blood infection (aOR 1.44, 95% CI 1.06, 1.96, P = 0.019) and acute placental infection (aOR 1.24, 95% CI 1.00, 1.54; 0.052) amongst primigravid women only, with corresponding trends observed when ID was used to describe maternal iron stores (Additional file 1: Tables S3). There was no evidence of effect modification of iron-malaria associations by malaria prevention regimen (Additional file 1: Tables S4) or enrolment Hb of ≤ 90 g/L, a trigger for enhanced iron-folate supplementation (Additional file 1: Tables S5).

## Discussion

The present study was conducted in a *P. falciparum* and *P. vivax* co-endemic setting of moderate transmission intensity. Peripheral blood malaria infection was detected in approximately 15% of women at their first antenatal visit and 6% at birth, and 19% of women had evidence of malaria infection on placental histology. The predominant malaria species infecting pregnant women was *P. falciparum* (80% of peripheral infections before study interventions and 60% at delivery, i.e. after drug allocation). ID, defined as an inflammation and infection-corrected ferritin level < 15 μg/L at first antenatal visit, was associated with reduced odds of peripheral infection at enrolment; reduced odds of peripheral and placental blood infection at birth; and reduced risk of past infection on placental histology. At antenatal enrolment, when the highest burden of peripheral infection was detected, ID was associated with a substantially reduced odds of *P. falciparum*, but not *P. vivax* infection, and a more pronounced decrease in microscopic than in submicroscopic infections. By contrast, ID was associated with reduced odds of peripheral *P. vivax* infection and submicroscopic infection at delivery. Associations between indicators of maternal iron stores and malaria infection at delivery were modified by gravidity. Amongst primigravid women only, there were increased odds of malaria infection (peripheral and placental blood infection, active infection on histology) associated with a two-fold increase in ferritin, and reduced odds of peripheral infection associated with ID. Collectively, these data provide further evidence in support of previously reported associations of indicators of maternal iron stores and the occurrence of peripheral and placental malaria infection during pregnancy [13, 16–18, 37, 38]. The data further suggest that associations may differ by parasite species, and are more pronounced when peripheral infections detectable by microscopy were considered.

How does an iron-replete state (or supplementation) increase the risk of malaria infection? Suppressed erythropoiesis in women with ID anaemia was associated with reduced growth rates of *P. falciparum* parasites in vitro, whilst their growth was enhanced in response to iron supplementation and consequential elevated erythropoiesis [39, 40]. Depleted antenatal iron stores were associated with reduced odds of *P. vivax* infection at delivery but not at enrolment. ID decreases production of reticulocytes, which *P. vivax* preferentially infects, so a protective effect might be envisaged. In addition to erythropoiesis-mediated impacts of ID on parasite growth, other mechanisms may be of relevance to iron-malaria interactions. Though ID may decrease susceptibility to disease it is worth acknowledging that it may also have a negative effect of the development of immunity. ID at the time of vaccination in young children is associated with decreased antibody to diphtheria, pertussis, and pneumococcal vaccines [41], and serum iron is positively associated with measles antibody titres in vaccinated individuals [42]. Experiments in animal models and in vitro suggest that ID causes defective B-cell proliferation and differentiation [42]. This is corroborated by recent reports of reduced levels of *P. falciparum*-specific antibodies in iron-deficient African children [43]. ID may thus affect the development and functionality of antibody features that can protect from malaria infection [44].

The size and the directionality of effects observed in our study were consistent with previous studies that assessed associations between ferritin and *P. falciparum* infection in pregnant women [13]. ID may hamper the development of immunity to pregnancy-associated malaria that translates into lower prevalence of placental infection and associated adverse birth outcomes in high transmission settings. In addition, ID may exert a protective effect in primigravid women by slowing parasite growth. In the present study, gravidity-dependent differences in associations of ID with malaria infection during pregnancy were observed. ID was associated with reduced odds of peripheral parasitaemia at delivery amongst primigravid women, and a two-fold increase in ferritin associated with malaria infection in primigravid but not multigravid women. Similar observations were made in earlier research from Tanzania. Iron-deficient primi- and secundigravidae had a lower prevalence of placental blood *P. falciparum* infection by light microscopy compared to their iron-replete counterparts. Amongst multigravidae, malaria prevalence did not differ significantly between iron-deficient and iron-replete women [16]. These data suggest that the development of gravidity-dependent protective immunity to placental infection [45] may modify the relationship between maternal ID and malaria protection. Malaria prevention approaches may also alter the association of ID and iron supplementation with malaria infection risk in pregnant women [20, 46]. In the present study, two markedly different malaria prevention approaches (a single dose of SPCQ at antenatal enrolment versus monthly IPTp with SPAZ) were used alongside insecticide treated bed nets,. However, there was no apparent effect modification of iron-malaria relationships by treatment arm. This suggests that in PNG both treatment regimens did not substantially alter malaria risk related to maternal iron stores.

A 2014 meta-analysis exclusively reported iron-malaria interactions when CRP-corrected ferritin levels, but not other markers, were used to determine maternal iron stores [13]. It may be that the effect of malaria-associated inflammation is not fully captured by CRP and AGP [47], which could confound the observed iron-malaria relationships. Whilst previous research, conducted in non-pregnant individuals, indicated a limited role of further correcting ferritin levels by concurrent malaria parasitaemia [36], foregoing correction of ferritin for both inflammation (CRP, AGP) *and* concurrent peripheral parasitaemia would have resulted in misclassification of 25% of women as iron replete in the present cohort, which prompted a primary analysis using ferritin levels corrected for both inflammation and malaria infection. However, associations between maternal ID or ferritin levels with malaria infection were highly comparable in analyses using ferritin levels corrected for CRP and AGP only (Additional file 1: Tables S1), and analyses using ferritin corrected for CRP, AGP and concurrent peripheral parasitaemia (Table 3). This suggests that correcting for presence of peripheral parasitaemia present at ferritin measurement has a limited role in confounding associations of ID and subsequent malaria infection during pregnancy, but may help with estimating true burden of ID in pregnancy. Mwangi et al. performed a similar analysis in Kenya, and showed that adjusting for CRP, AGP and parasitaemia gave a better fit for their model to predict ID [21]. Whether placental infections without detectable blood stream infection [48] affect ferritin levels is unknown, as is whether such occult infections are associated with increases in ferritin levels that are not captured by adjustment for CRP or AGP. More recent reports of associations between other markers of iron stores (transferrin saturation, soluble transferrin receptor [sTFR]/log_10_ ferritin ratios) and malaria in pregnant women and children [37, 49] support the notion of a true biological effect. This is further consolidated by prospective studies reporting increased risk of malaria infection in pregnant women and children receiving iron supplements in absence of concurrent anti-malarial chemoprevention [12, 46]. However, trials have demonstrated that iron supplementation in pregnancy is safe when IPTp-SP and bed nets are used concurrently [20, 22].

The strengths of the present research include comprehensive assessments of infection status in a large cohort of pregnant women at risk of both *P. falciparum* and *P. vivax* infection; adjustments of ferritin levels for concurrent inflammation and peripheral parasitaemia; and adjustments for potential confounders including maternal residence, a marker of infection exposure risk. Weaknesses include that the provision and uptake of iron supplementation was unmonitored and may have differed between iron deficient and iron replete women. Furthermore, residual confounding of ferritin-malaria interactions by the presence of subpatent placental infections at the time of ferritin measurement could not be ruled out.

## Conclusions

There are substantial associations between markers of antenatal iron status and malaria infection during pregnancy. Whilst residual confounding due to misclassification of ID and unmeasured predictors of malaria infection cannot be ruled out, maternal iron status may influence malaria risk by altering parasite growth potential and maternal immune responses to malarial infection, including placental malaria. Prospective studies that assess the temporal relationships between pre-pregnancy and early pregnancy iron stores and iron supplementation during pregnancy with malaria incidence and the development of anti-malarial immunity could consolidate our understanding of iron-malaria relationships in pregnancy, alongside evaluations of the impact of placental infection on markers of maternal iron status. Broader validation of the approach to adjust ferritin levels for both inflammation and *Plasmodium* infection against reference methods to diagnose ID, such as bone marrow iron stores, are desirable in the future.

## Supplementary Information

**Additional file 1: Table S1.** Association of maternal ferritin levels at antenatal enrolment with malaria infection at antenatal enrolment and at delivery, Madang, Papua New Guinea, 2009–2013. Ferritin levels were adjusted for concurrent inflammation (C-reactive protein and α-1-acid glycoprotein). **Table S2.** Association of ferritin levels at antenatal enrolment with malaria infection at delivery in women without peripheral *Plasmodium* parasitaemia at antenatal enrolment, Madang, Papua New Guinea, 2009–2013. Ferritin levels were adjusted for concurrent inflammation (C-reactive protein and α-1-acid glycoprotein). **Table S3.** Associations between maternal iron status (ferritin) at first antenatal visit and malaria infection at delivery, stratified by gravidity, Madang Province, Papua New Guinea, 2009–2013. **Table S4.** Associations between maternal iron status (ferritin) at first antenatal visit and malaria infection at delivery, stratified by malaria prevention regimen, Madang Province, Papua New Guinea, 2009–2013. **Table S5.** Associations between maternal iron status (ferritin) at first antenatal visit and malaria infection at delivery, stratified by haemoglobin status at antenatal enrolment, Madang Province, Papua New Guinea, 2009–2013.

## Data Availability

Data are available from the WWARN data repository (http://www.wwarn.org/working-together/sharing-data/accessing-data) for researchers who meet the criteria for access to confidential data and from the corresponding author on reasonable request.

## Abbreviations

AGP: α-1-Acid glycoprotein
CI: Confidence interval
CRP: C-reactive protein
Hb: Haemoglobin
ID: Iron deficiency
IPTp: Intermittent preventive treatment in pregnancy
IQR: Interquartile range
OR: Odds ratio
PNG: Papua New Guinea
PCR: Polymerase chain reaction
SD: Standard deviation
