# Supplementary material for: Associations of maternal iron deficiency with malaria infection in a cohort of pregnant Papua New Guinean women
Source: Malar J. 2022 May 26;21:153. doi: 10.1186/s12936-022-04177-8 (PMC9137066; doi:10.1186/s12936-022-04177-8)
Supplement: Supplementary file 1 — Additional file 1: Table S1. Association of maternal ferritin levels at antenatal enrolment with malaria infection at antenatal enrolment and at delivery, Madang, Papua New Guinea, 2009–2013. Ferritin levels were adjusted for concurrent inflammation (C-reactive protein and α-1-acid glycoprotein). Table S2. Association of ferritin levels at antenatal enrolment with malaria infection at delivery in women without peripheral Plasmodium parasitaemia at antenatal enrolment, Madang, Papua New Guinea, 2009–2013. Ferritin levels were adjusted for concurrent inflammation (C-reactive protein and α-1-acid glycoprotein). Table S3. Associations between maternal iron status (ferritin) at first antenatal visit and malaria infection at delivery, stratified by gravidity, Madang Province, Papua New Guinea, 2009–2013. Table S4. Associations between maternal iron status (ferritin) at first antenatal visit and malaria infection at delivery, stratified by malaria prevention regimen, Madang Province, Papua New Guinea, 2009–2013. Table S5. Associations between maternal iron status (ferritin) at first antenatal visit and malaria infection at delivery, stratified by haemoglobin status at antenatal enrolment, Madang Province, Papua New Guinea, 2009–2013. [file 12936_2022_4177_MOESM1_ESM.docx]

**Supplemental Table 1.** Association of maternal ferritin levels at antenatal enrolment with malaria infection at antenatal enrolment and at delivery, Madang, Papua New Guinea, 2009-2013. Ferritin levels were adjusted for concurrent inflammation (C-reactive protein and α-1-acid glycoprotein).

| **Factor** | | **AOR (95% CI)** | **P value** |
| --- | --- | --- | --- |
| **Enrolment** | |  |  |
| *Peripheral infection** | |  |  |
| Iron deficiency | |  |  |
|  | Ferritin <15 μg/L | 0.51 (0.38, 0.67) | <0.001 |
|  | Ferritin ≥15 μg/L | Reference |  |
| Log_2_(ferritin) | | 1.37 (1.23, 1.53) | <0.001 |
|  | |  |  |
| **Delivery** | |  |  |
| *Peripheral infection** | |  |  |
| Iron deficiency | |  |  |
|  | Ferritin <15 μg/L | 0.51 (0.34, 0.76) | 0.001 |
|  | Ferritin ≥15 μg/L | Reference |  |
| Log_2_(ferritin) | | 1.28 (1.11, 1.49) | 0.001 |
|  | |  |  |
| Placental infection (blood)* | |  |  |
| Iron deficiency | |  |  |
|  | Ferritin <15 μg/L | 0.76 (0.44, 1.31) | 0.32 |
|  | Ferritin ≥15 μg/L | Reference |  |
| Log_2_(ferritin) | | 1.23 (1.00, 1.51) | 0.048 |
|  | |  |  |
| Placental infection (histology)* | |  |  |
| Iron deficiency | |  |  |
|  | Active infection | 0.90 (0.58, 1.38) | 0.62 |
|  | Past infection | 0.37 (0.25, 0.54) | <0.001 |
|  | No infection | Reference |  |
|  |  |  |  |
| Log_2_(ferritin) | |  |  |
|  | Active infection | 1.04 (0.88, 1.23) | 0.64 |
|  | Past infection | 1.63 (1.40, 1.90) | <0.001 |
|  | No infection | Reference |  |

**Note.** Analyses adjusted for gravidity, maternal age, malaria chemoprevention regimen, rural location, and gestational age at ferritin measurement. Ferritin levels were adjusted for concurrent inflammation (C-reactive protein and α-1-acid glycoprotein) using the BRINDA (Biomarkers Reflecting Inflammation and Nutritional Determinants of Anemia) approach [36].

Abbreviations: AOR, adjusted odds ratio; CI, confidence interval

*Include 1,886, 1,886, 1,253 and 1,356 women with malaria infection data (*Plasmodium falciparum*, *P. vivax*) from peripheral blood at enrolment, peripheral blood at delivery, placental blood, and placental histology collected at delivery, respectively.

**Supplemental Table 2.** Association of ferritin levels at antenatal enrolment with malaria infection at delivery in women without peripheral *Plasmodium* parasitaemia at antenatal enrolment, Madang, Papua New Guinea, 2009-2013. Ferritin levels were adjusted for concurrent inflammation (C-reactive protein and α-1-acid glycoprotein).

| **Factor** | | **AOR (95% CI)** | **P value** |
| --- | --- | --- | --- |
|  | |  |  |
| *Peripheral infection** | |  |  |
| Iron deficiency | |  |  |
|  | Ferritin <15 μg/L | 0.60 (0.38, 0.96) | 0.032 |
|  | Ferritin ≥15 μg/L | Reference |  |
| Log_2_(ferritin) | | 1.27 (1.06, 1.51) | 0.009 |
|  | |  |  |
| Placental infection (blood)* | |  |  |
| Iron deficiency | |  |  |
|  | Ferritin <15 μg/L | 0.83 (0.45, 1.55) | 0.56 |
|  | Ferritin ≥15 μg/L | Reference |  |
| Log_2_(ferritin) | | 1.28 (1.00, 1.63) | 0.053 |
|  | |  |  |
| Placental infection (histology)* | |  |  |
| Iron deficiency | |  |  |
|  | Active infection | 1.02 (0.64, 1.62) | 0.94 |
|  | Past infection | 0.45 (0.28, 0.72) | 0.001 |
|  | No infection | Reference |  |
|  |  |  |  |
| Log_2_(ferritin) | |  |  |
|  | Active infection | 0.96 (0.80, 1.41) | 0.62 |
|  | Past infection | 1.39 (1.15, 1.68) | 0.001 |
|  | No infection | Reference |  |

**Note.** Analyses adjusted for gravidity, maternal age, malaria chemoprevention regimen, rural location, and gestational age at ferritin measurement. Ferritin levels were adjusted for concurrent inflammation (C-reactive protein and α-1-acid glycoprotein) using the BRINDA (Biomarkers Reflecting Inflammation and Nutritional Determinants of Anemia) approach [36].

Abbreviations: AOR, adjusted odds ratio; CI, confidence interval

*Include 1,627, 1077 and 1,171 women with malaria infection data (*Plasmodium falciparum*, *P. vivax*) from peripheral blood at delivery, placental blood, and placental histology collected at delivery, respectively.

**Supplemental Table 3.** Associations between maternal iron status (ferritin) at first antenatal visit and malaria infection at delivery, stratified by gravidity, Madang Province, Papua New Guinea, 2009-2013.

|  | | **Gravidity** | |  |
| --- | --- | --- | --- | --- |
|  | | *Primigravida*  **n/N (%) or AOR (95% CI); *p*** | *Multigravida*  **n/N (%) or AOR (95% CI); *p*** | *p* interaction parameter |
| *Peripheral infection****** | | 62/931 (6.7) | 58/957 (6.0) |  |
| *Iron deficiency* | |  |  |  |
|  | Ferritin <15 μg/L | 0.44 (0.25, 0.76), 0.003 | 1.12 (0.61, 2.05), 0.72 | 0.018 |
|  | Ferritin ≥15 μg/L | Reference | Reference |  |
|  |  |  |  |  |
|  | Log_2_(ferritin) | 1.51 (1.23, 1.84), <0.001 | 1.04 (0.83, 1.29), 0.76 | 0.010 |
|  | |  |  |  |
| *Placental infection (blood)****** | | 30/621 (4.8) | 33/632 (5.2) |  |
| *Iron deficiency* | |  |  |  |
|  | Ferritin <15 μg/L | 0.67 (0.47, 1.23); 0.12 | 1.21 (0.63, 2.32); 0.57 | 0.14 |
|  | Ferritin ≥15 μg/L | Reference | Reference |  |
|  | |  |  |  |
|  | Log_2_(ferritin) | 1.44 (1.06, 1.96); 0.019 | 1.03 (0.78, 1.36); 0.83 | 0.056 |
|  | |  |  |  |
| *Active placental infection (histology)******** | | 58/590 (9.8) | 45/615 (7.3) |  |
| *Iron deficiency* | |  |  |  |
|  | Ferritin <15 μg/L | 0.68 (0.38, 1.21); 0.19 | 1.45 (0.68, 3.09), 0.34 | 0.074 |
|  | Ferritin ≥15 μg/L | Reference | Reference |  |
|  | |  |  |  |
|  | Log_2_(ferritin) | 1.24 (1.00, 1.54); 0.052 | 0.81 (0.62, 1.05); 0.10 | 0.009 |
|  | |  |  |  |
| *Past placental infection (histology)******** | | 97/629 (15.4) | 54/624 (8.7) |  |
| *Iron deficiency* | |  |  |  |
|  | Ferritin <15 μg/L | 0.35 (0.22, 0.56); <0.001 | 0.35 (0.19, 0.64); <0.001 | 0.91 |
|  | Ferritin ≥15 μg/L | Reference | Reference |  |
|  | |  |  |  |
|  | Log_2_(ferritin) | 1.55 (1.28, 1.86); <0.001 | 1.82 (1.41, 2.34); <0.001 | 0.27 |

**Note.** Analyses adjusted for maternal age, malaria chemoprevention regimen, rural location, and gestational age at ferritin measurement. Ferritin levels were adjusted for concurrent inflammation and peripheral malaria parasitaemia at antenatal enrolment using the BRINDA (Biomarkers Reflecting Inflammation and Nutritional Determinants of Anemia) approach [36]. Abbreviations: AOR, adjusted odds ratio; CI, confidence interval

*Include 1,886, 1,253 and 1,356 women with malaria data from peripheral blood, placental blood, and placental histology collected at delivery, respectively.

**Supplemental Table 4.** Associations between maternal iron status (ferritin) at first antenatal visit and malaria infection at delivery, stratified by malaria prevention regimen, Madang Province, Papua New Guinea, 2009-2013.

|  | | **Malaria prevention regimen** | |  |
| --- | --- | --- | --- | --- |
|  | | *Sulphadoxine-pyrimethamine plus chloroquine* | *Sulphadoxine-pyrimethamine plus azithromycin* | *p* interaction parameter |
|  | | **n/N (%) or AOR (95% CI); *p*** | **n/N (%) or AOR (95% CI); *p*** |  |
| **Delivery*** | |  |  |  |
| *Peripheral infection** | | 76/934 (8.1) | 44/945 (4.7) |  |
| Iron deficiency μg/L | |  |  |  |
|  | Ferritin <15 μg/L | 0.86 (0.52, 1.43); 0.57 | 0.46 (0.24, 0.86); 0.016 | 0.17 |
|  | Ferritin ≥15 μg/L | Reference | Reference |  |
|  | |  |  |  |
|  | Log_2_(ferritin) | 1.19 (0.99, 1.43); 0.066 | 1.44 (1.13, 1.83); 0.003 | 0.27 |
|  | |  |  |  |
| *Placental infection (blood)** | | 41/622 (6.6) | 22/631 (3.5) |  |
| *Iron deficiency μg/L* | |  |  |  |
|  | Ferritin <15 μg/L | 0.91 (0.46, 1.81); 0.78 | 0.36 (0.14, 0.92); 0.033 | 0.084 |
|  | Ferritin ≥15 μg/L | Reference | Reference |  |
|  | |  |  |  |
|  | Log_2_(ferritin) | 1.19 (0.93, 1.52); 0.16 | 1.33 (0.91, 1.93); 0.14 | 0.57 |
|  | |  |  |  |
| Active placental infection (histology)* | | 64/606 (10.6) | 39/599 (6.5) |  |
|  | Ferritin <15 μg/L | 0.93 (0.52, 1.68); 0.81 | 0.86 (0.43, 1.75), 0.68 | 0.89 |
|  | Ferritin ≥15 μg/L | Reference | Reference |  |
|  | |  |  |  |
|  | Log_2_(ferritin) | 1.13 (0.92, 1.39); 0.23 | 0.89 (0.68, 1.17); 0.41 | 0.16 |
|  | |  |  |  |
| Past placental infection (histology)* | | 74/616 (12.0) | 77/637 (12.1) |  |
|  | Ferritin <15 μg/L | 0.35 (0.21, 0.59); <0.001 | 0.34 (0.20, 0.59); <0.001 | 0.80 |
|  | Ferritin ≥15 μg/L | Reference | Reference |  |
|  | |  |  |  |
|  | Log_2_(ferritin) | 1.62 (1.32, 1.99); <0.001 | 1.69 (1.35, 2.11); <0.001 | 0.69 |

**Note.** Analyses adjusted for gravidity, maternal age, rural location, and gestational age at ferritin measurement. Ferritin levels were adjusted for concurrent inflammation and peripheral malaria parasitaemia at antenatal enrolment using the BRINDA (Biomarkers Reflecting Inflammation and Nutritional Determinants of Anemia) approach [36]. Abbreviations: AOR, adjusted odds ratio; CI, confidence interval

*Include 1,886, 1,253 and 1,356 women with malaria data from peripheral blood, placental blood, and placental histology collected at delivery, respectively.

**Supplemental Table 5.** Associations between maternal iron status (ferritin) at first antenatal visit and malaria infection at delivery, stratified by haemoglobin status at antenatal enrolment, Madang Province, Papua New Guinea, 2009-2013.

|  | | **Haemoglobin level at enrolment** | |  |
| --- | --- | --- | --- | --- |
|  | | *≤90 g/L* | *>90 g/L* | *p* interaction parameter |
|  | | **n/N (%) or AOR (95% CI); *p*** | **n/N (%) or AOR (95% CI); *p*** |  |
| **Delivery*** | |  |  |  |
| *Peripheral infection** | | 40/527 (7.6) | 76/1,285 (5.9) |  |
| Iron deficiency μg/L | |  |  |  |
|  | Ferritin <15 μg/L | 0.66 (0.34, 1.29); 0.22 | 0.66 (0.40, 1.08); 0.099 | 0.51 |
|  | Ferritin ≥15 μg/L | Reference | Reference |  |
|  | |  |  |  |
|  | Log_2_(ferritin) | 1.17 (0.93, 1.47); 0.19 | 1.37 (1.13, 1.67); 0.001 | 0.66 |
|  | |  |  |  |
| *Placental infection (blood)** | | 16/345 (4.6) | 40/848 (4.7) |  |
| *Iron deficiency μg/L* | |  |  |  |
|  | Ferritin <15 μg/L | 0.90 (0.31, 2.65); 0.85 | 0.66 (0.33, 1.33); 0.25 | 0.79 |
|  | Ferritin ≥15 μg/L | Reference | Reference |  |
|  | |  |  |  |
|  | Log_2_(ferritin) | 1.10 (0.77, 1.57); 0.59 | 1.22 (0.93, 1.60); 0.15 | 0.88 |
|  | |  |  |  |
| Active placental infection (histology)* | | 39/284 (13.7) | 57/867 (6.6) |  |
|  | Ferritin <15 μg/L | 0.63 (0.29, 1.35); 0.23 | 0.97 (0.53, 1.77), 0.92 | 0.35 |
|  | Ferritin ≥15 μg/L | Reference | Reference |  |
|  | |  |  |  |
|  | Log_2_(ferritin) | 1.29 (0.98, 1.66); 0.066 | 0.99 (0.79, 1.24); 0.91 | 0.13 |
|  | |  |  |  |
| Past placental infection (histology)* | | 73/318 (23.0) | 72/882 (8.2) |  |
|  | Ferritin <15 μg/L | 0.27 (0.16, 0.45); <0.001 | 0.30 (0.16, 0.55); <0.001 | 0.86 |
|  | Ferritin ≥15 μg/L | Reference | Reference |  |
|  | |  |  |  |
|  | Log_2_(ferritin) | 1.88 (1.48, 2.38); <0.001 | 1.81 (1.45, 2.26); <0.001 | 0.51 |

**Note.** Analyses adjusted for gravidity, maternal age, rural location, and gestational age at ferritin measurement. Ferritin levels were adjusted for concurrent inflammation and peripheral malaria parasitaemia at antenatal enrolment using the BRINDA (Biomarkers Reflecting Inflammation and Nutritional Determinants of Anemia) approach [36]. Abbreviations: AOR, adjusted odds ratio; CI, confidence interval

*Include 1,809, 1,192 and 1,296 women with complete haemoglobin at enrolment and malaria data from peripheral blood, placental blood, and placental histology collected at delivery, respectively.
